# Sonodynamic Treatment Induces Selective Killing of Cancer Cells in an In Vitro Co-Culture Model

**DOI:** 10.3390/cancers13153852

**Published:** 2021-07-30

**Authors:** Federica Foglietta, Vanessa Pinnelli, Francesca Giuntini, Nadia Barbero, Patrizia Panzanelli, Gianni Durando, Enzo Terreno, Loredana Serpe, Roberto Canaparo

**Affiliations:** 1 Department of Drug Science and Technology, University of Torino, 10125 Torino, Italy; federica.foglietta@unito.it (F.F.); vanessapinnelli705@gmail.com (V.P.); roberto.canaparo@unito.it (R.C.); 2 School of Pharmacy and Biomolecular Sciences, Liverpool John Moores University, Liverpool L3 3AF, UK; f.giuntini@ljmu.ac.uk; 3 Department of Chemistry, NIS Interdepartmental Centre and INSTM Reference Centre, University of Torino, 10125 Torino, Italy; nadia.barbero@unito.it; 4 Department of Neuroscience Rita Levi Montalcini, University of Torino, 10125 Torino, Italy; patrizia.panzanelli@unito.it; 5 National Institute of Metrological Research (INRIM), 10135 Torino, Italy; g.durando@inrim.it; 6 Department of Molecular Biotechnology and Health Sciences, University of Torino, 10126 Torino, Italy; enzo.terreno@unito.it

**Keywords:** ultrasound, sonodynamic therapy, porphyrin, cancer cells, membrane fluidity

## Abstract

**Simple Summary:**

A review of over six decades of cancer chemotherapies, including recent immunotherapy, reveals partial success in the battle against cancer. One of the main reasons for this slow progress is the failure, by mainstream anticancer treatments, to distinguish between cancer cells and normal cells. For this reason, the aim of our study was to assess if sonodynamic therapy (SDT), a new anticancer approach, can affect cancer cells only, avoiding any harmful effects on normal cells. SDT aims to cure malignant tumors by using a chemical compound (sonosensitizer) triggered by ultrasound exposure. For this purpose, the effects on cancer cells and normal cells, namely HT-29 cells and HDF 106-05 cells, subjected to sonodynamic treatment were investigated. Our results show that, according to different plasma membrane properties of cancer cells and normal cells, a different sonodynamic effect occurs, reaching a remarkable cytotoxic effect on cancer cells only.

**Abstract:**

Sonodynamic Therapy (SDT) is a new anticancer strategy based on ultrasound (US) technique and is derived from photodynamic therapy (PDT); SDT is still, however, far from clinical application. In order to move this therapy forward from bench to bedside, investigations have been focused on treatment selectivity between cancer cells and normal cells. As a result, the effects of the porphyrin activation by SDT on cancer (HT-29) and normal (HDF 106-05) cells were studied in a co-culture evaluating cell cytotoxicity, reactive oxygen species (ROS) production, mitochondrial function and plasma membrane fluidity according to the bilayer sonophore (BLS) theory. While PDT induced similar effects on both HT-29 and HDF 106-05 cells in co-culture, SDT elicited significant cytotoxicity, ROS production and mitochondrial impairment on HT-29 cells only, whereas HDF 106-05 cells were unaffected. Notably, HT-29 and HDF 106-05 showed different cell membrane fluidity during US exposure. In conclusion, our data demonstrate a marked difference between cancer cells and normal cells in co-culture in term of responsiveness to SDT, suggesting that this different behavior can be ascribed to diversity in plasma membrane properties, such as membrane fluidity, according to the BLS theory.

## 1. Introduction

Cancer is a major public health problem worldwide and therefore, continuous scientific efforts in this field are clearly needed [1]. Therefore, in recent decades, thanks mainly to increasing knowledge on cancer molecular biology and growing interest in applying bio- and nano-technology techniques against this pathology, cancer therapeutic strategies have notably changed [2,3]. However, despite these advancements, the overall cancer death rate still remains high as the critical issue of targeting cancer cells with high selectivity has not been reached for most cancers [4]. For this reason, novel and challenging therapeutic approaches must be explored to improve selectivity for cancer cells over normal cells to avoid systemic toxicity and to overcome drug resistance [5].

In order to investigate new therapeutic anticancer approaches able to improve cancer selectivity, we have investigated sonodynamic therapy (SDT), a therapeutic strategy derived from photodynamic therapy (PDT), based on the synergistic effect triggered by combining a suitable chemical compound (sonosensitizer) and low-intensity ultrasound (US), which is used to kill cancer cells and microbial cells [6,7]. The anticancer effectiveness of SDT has been demonstrated in in vitro and in vivo animal models [8,9,10,11]. Despite the promising results of this approach, however, only a few clinical reports have described the use of SDT [6]. In our opinion, for SDT to be quickly turned into the clinical setting, a deeper understanding of its mechanism of action and its potentiality is needed. Therefore, the aim of our work was to shed new light on the SDT mechanism of action and to understand if selectivity towards cancer could be one of the main features of this approach. To this end, in our previous investigations, different outcomes of cancer cells and normal cells to SDT were reported, supporting the finding that cancer cells and normal cells behave differently in response to SDT [12]. Indeed, it has been suggested that cell responsiveness to US is different between cancer cells and normal cells as the resistance of these cell types to physical stress is not the same [13]. Moreover, recently, Geltmeier and colleagues reported that a tension/compression force of US can provoke damage preferentially on cancer cells, compared to normal cells [14].

To achieve our goals, SDT experiments were carried out in cancer and normal cell lines, namely the human colon cancer HT-29 cell line and the dermal fibroblast HDF 106-05 cell line, in co-culture to provide a more representative in vivo-like tissue model [15] and to avoid any misinterpretation of the results when the same experiments are performed separately.

Since the generation of reactive oxygen species (ROS) is considered a pivotal step in SDT anticancer activity, we performed our STD experiments with a palladium (II) porphyrin complex (Pd-P) as a sonosensitizer because, under US exposure, it resulted in being the most efficient sonosensitizer, compared to other metal–porphyrin complexes such as iron (III) and zinc (II) porphyrin complexes, in generating singlet oxygen (^1^O_2_) and hydroxyl radicals (^•^OH), and in US-mediated cancer cell killing [12]. Therefore, the selective cytotoxicity of SDT towards cancer HT-29 cells co-cultured with normal HDF 106-05 cells was evaluated in terms of cell death and ROS generation. In addition, we tried to elucidate the SDT mechanism of action by investigating the possible process responsible for selective responsiveness of cancer cells to SDT treatment. Firstly, we hypothesized that the intracellular activation of the sonosensitizer was probably due to a US-mediated energy transfer in the form of intramembrane cavitation across the cytoplasmic membrane according to the bilayer sonophore (BLS) theory, suggesting that the cell type-dependent responsiveness to SDT could rely on specific plasma membrane features. Secondly, we hypothesized that mitochondria were involved as the main intracellular target of SDT in the cell type-dependent SDT response [16,17]. For this reason, the plasma membrane diversity between cancer cells and normal cells was investigated in terms of SDT impact on plasma membrane fluidity, while the mitochondrial membrane potential was studied to confirm the pivotal role of these intracellular organelles in the efficacy of SDT.

## 2. Materials and Methods

### 2.1. Preparation of Porphyrin-Metal Complex Solution

5,10,15,20-tetrakis(*N*-methylpyridinium-4-yl) porphyrinato palladium (II) tetrachloride (Pd-P) was synthesized according to the method described by Giuntini et al. [12]. In order to obtain a 2 mM solution, 9.2 mg of Pd-P powder (MW 925.05 g/mol) was reconstituted in 5 mL cell medium without serum that was then filtered with sterile syringe filter membrane 0.2 μm (Enrico Bruno S.r.l., Torino, Italy) and stored in 1 mL aliquots at −20 °C away from the light.

### 2.2. Cell Culture

The human Caucasian colon adenocarcinoma cell line, HT-29 (Interlab Cell Line Collection, Genova, Italy), was cultured as a monolayer in RPMI-1640 growth medium (Sigma-Aldrich, Milano, Italy). The normal adult human primary dermal fibroblast cell line, HDF 106-05 (ECACC, Salisbury, UK), was cultured as a monolayer in DMEM growth medium (Sigma-Aldrich). Both cell culture media were supplemented with 10% decomplemented fetal bovine serum (FBS) (Lonza, Verviers, Belgium), 2 mM-glutamine, 100 UI/mL penicillin and 100 μg/mL streptomycin antibiotic (Sigma-Aldrich). Both cell lines were maintained in a dark incubator (Thermo Fisher Scientific, Waltham, MA, USA) in a humidified atmosphere containing 5% CO_2_ at 37 °C.

#### HT-29 and HDF 106-05 Cell Co-Culture

For the co-culture sonodynamic and photodynamic experiments, equal amounts of HT-29 and HDF 106-05 cells were used (1:1), with a final concentration of 5 × 10^5^ cells. In particular, HT-29 and HDF 106-05 cells were maintained in co-culture with an equal mix of the respective cell culture medium.

### 2.3. Intracellular Porphyrin Fluorescence Quantification

According to our previous work [12], the non-cytotoxic Pd-P concentration required to perform sonodynamic and photodynamic treatment on HT-29 and HDF 106-05 cells was 500 µM for P-Pd. In order to ascertain the correct time of sonosensitizer incubation before performing sonodynamic and photodynamic treatment, semiquantitative intracellular uptake of Pd-P in HT-29 and HDF 106-05 cells was investigated using the fluorescence plate reader EnSight (PerkinElmer, Waltham, MA, USA). Briefly, 5 × 10^3^ HT-29 and HDF 106-05 cells were cultured in total black 96-well plates and, 24 h after seeding, cells were incubated for 1, 6, 12 and 24 h with Pd-P at 500 μM, respectively. Prior to analysis, culture media was removed, cells were washed with PBS, and nuclei were stained with 4′, 6-diamidino-2-phenylindole (DAPI) 2 μL/mL for 10 min, then fixed with paraformaldehyde (PAF) 4% solution (Sigma-Aldrich) for 10 min at room temperature. Each well was then supplemented with 200 µL PBS for plate reading according to the plate normalization procedure for top reading setting.

DAPI is a blue, fluorescent, nucleic acid stain that preferentially stains double-stranded DNA. The detected fluorescence is directly proportional to the amount of DNA present in the cell, and as DAPI is rapidly taken up into cellular DNA, it is easy to discriminate fluorescent nuclei with no detectable cytoplasmic fluorescence. Fluorescence was read according to the parameters derived from relative spectra: λ_ex_ 358 nm–λ_em_ 461 nm for DAPI and λ_ex_ 416 nm–λ_em_ 683 nm for P-Pd. DAPI fluorescence from nuclei staining was used to normalize the fluorescence signal from the plate reader according to the cell number in each well. Porphyrin uptake was expressed as Intensity of Fluorescence DAPI Related Index (IFDR).

### 2.4. Confocal Microscopy

Qualitative uptake of Pd-P in HT-29 and HDF 106-05 cells was performed by confocal microscopy to investigate its intracellular localization. Specifically, confocal porphyrins uptake was performed at 24 h after the incubation. A total of 1 × 10^5^ HT-29 and HDF 106-05 cells were seeded in 24-well plate with glass coverslips on the bottom, and 24 h after the seeding, cells were then incubated with Pd-P (500 μM) for 24 h. Cells on slides were fixed with 4% PAF (Sigma-Aldrich) for 15 min at room temperature. Cells were washed, and glass coverslips were placed on slides with Fluoroshield (Sigma-Aldrich) mounting medium for preserving and preventing rapid photobleaching of the fluorescent probe.

Confocal images were acquired using a laser scanning (λ_ex_ 405 nm and λ_em_ 633 nm) confocal microscope (Zeiss LSM5 Pascal, Oberkochen, Germany) with a 40× oil immersion objective using the multi-track mode. Images were analyzed with ImageJ software (FiJi, Bristol, UK, version 2.0). In order to quantify the porphyrin fluorescence in HT-29 and HDF 106-05 cells obtained by confocal acquisitions, five cells per each sample in three independent regions of the slide were considered, and the following formula for the corrected total cell fluorescence (CTCF), calculated with Image J, was then used [18]: CTCF = integrated density − (area of selected cell × mean fluorescence of background readings).

Data are expressed as CTCF mean of three independent regions.

### 2.5. GSH Evaluation

The total glutathione level, glutathione disulfide (GSSG) and reduced glutathione (GSH) of HT-29 and HDF 106-05 as single cell lines was determined using the Glutathione Assay Kit (Sigma-Aldrich), according to the manufacturer’s instructions. The GSH content (nmol) was normalized to protein content in each sample by quantifying cell protein concentration (μg/mL) using the Quant-iT Protein Assay Kit by using the fluorimeter Qubit (Invitrogen-Life Technologies, Milano, Italy). Calibration was performed by applying a two-point standard curve, according to the manufacturer’s instructions. Briefly, GSH reacts with 5,5′-dithiobis (2-nitrobenzoic acid) (DTNB) in a recycling assay and produces GSSG and the 1,3,5-trinitrobenzene (TNB) anion, which can be detected by absorbance. In turn, the enzyme glutathione reductase then reduces GSSG, which releases GSH that can react with another DTNB molecule. Therefore, the rate of TNB production is measured rather than a single determination of how much DTNB reacts with GSH, as it is proportional to the initial amount of GSH [8]. The plate was read at 412 nm on a microplate reader Asys UV 340 (Biochrom, Cambridge, UK), and the amount of GSH was expressed in nmol/µg protein.

### 2.6. Sonodynamic Treatment

HT-29 and HDF 106-05 cells in the exponential growth phase were preincubated in the dark for 24 h with the sonosensitizer, Pd-P (500 μM), in 6-well plates. For the co-culture experiments, cells were then washed with PBS, trypsinized with 0.05% trypsin—0.02% EDTA solution and normalized to 5.0 × 10^5^ cells consisting of an equal amount of HT-29 cells and HDF 106-05 cells (1:1) in 2.5 mL PBS in polystyrene tubes (TPP, Trasadingen, Switzerland) away from the light. The polystyrene tube (10 mm diameter) containing the cell suspension was connected to the US transducer by means of a specific mechanical adaptor, with a chamber filled with temperature controlled ultrapure water, in order to fix the distance from the transducer at 17 mm, to control the temperature variance and to have reproducible measurement conditions [19].

US field was generated using a piezoelectric plane wave transducer (25.4 mm diameter), which operates in continuous wave mode at a frequency of 1.866 MHz, connected to a function generator (Type 33250; Agilent, Santa Clara, CA, USA) and a power amplifier (Type AR 100A250A; Amplifier Research, Souderton, PA, USA). All the conditions were then exposed to US generated at a 1.5 W/cm^2^ intensity for 5 min. An important aspect to be taken into consideration during US exposure is temperature control to avoid the hyperthermic effect. Therefore, the temperature of the cell suspension exposed to US was monitored by a thermocouple sensor (RS Components, Milano, Italy), and the maximum temperature recorded during the experiments in the US exposed samples was below 29 °C.

### 2.7. Ultrasound Field Characterization

US intensity (I) was measured as I = P/A where P is the US beam power and A is the cross-sectional area of US beam (A). The standardized method used to measure P is based on the radiation force balance principle, which is defined as the time averaged force exerted by an acoustic field on a target that intercepts the US beam. The measuring system was developed by the National Institute of Metrological Research (INRIM, Torino, Italy) and is based on a commercial balance (Mettler Toledo, model SAG-285, Columbus, OH, USA). Moreover, the spatial distribution of the US pressure has been evaluated by a scanning tank system (Onda Corp., Sunnyvale, CA, USA) with needle hydrophone (HNA-0400, Onda Corp.) and a preamplifier (AH-2020, Onda Corp.). During the 5 min insonation process at our experimental parameters, into the polystyrene tube, a value of maximum root-mean-square (rms) acoustic pressure of 300 kPa was measured.

### 2.8. Cytotoxicity Evaluation

At the end of the sonodynamic treatment, 8000 cells were plated in 24-well culture plates and then manually counted at 24, 48 and 72 h after the treatment, considering five different regions per well. Cytotoxicity was expressed as percentage (%) according to the following equation: % cytotoxicity = 100 × (untreated cell number − treated cell number).

### 2.9. Photodynamic Treatment

HT-29 and HDF 106-05 cells in the exponential growth phase were preincubated in the dark for 24 h with the sonosensitizer, Pd-P (500 μM), in 6-well plates. For the co-culture experiments, cells were then washed with PBS, trypsinized with 0.05% trypsin—0.02% EDTA solution and normalized to 5.0 × 10^5^ cells consisting of an equal amount of colon cancer cells and fibroblast cells (1:1) in 2.5 mL PBS in polystyrene tubes (TPP) protected from the light. The light-emitting source of the system is based on InGaN light-emitting diodes (Cree Inc, Durham, NC, USA) with 20 mW maximum radiant power (emitted flux), and a central wavelength of 405 nm. The system allows continuous radiant flux regulation from 0 to 20 mW, with a programmable non-switching diode current source and energy fluency rates were adjusted to 15 mW/cm^2^. In particular, cell suspensions were illuminated for 5 min in a dark box. Following the photodynamic treatment, 8000 cells were plated in 24-well culture plates, and then manually counted at 24, 48 and 72 h after the treatment, considering five different regions per well. Cytotoxicity was expressed as a percentage according to the following equation: % cytotoxicity = 100 × (untreated cell number − treated cell number).

### 2.10. Flow Cytometry Assay

Cytofluorimetric assays were performed using a C6 flow cytometer (Accuri Cytometers, Annarbor, MI, USA) in order to investigate intracellular ROS production and mitochondrial membrane potential.

#### 2.10.1. Intracellular ROS Production

Intracellular ROS generation after sonodynamic and photodynamic treatment in HT-29 or HDF 106-05 cells was investigated by using the 2,7-dichlorodihydrofluorescein diacetate (DCFH-DA; Sigma-Aldrich) probe. Briefly, cells in the exponential growth phase were incubated with Pd-P 500 μM for 24 h, and 10 µM DCFH-DA for the last 30 min of incubation at 37 °C protected from light. Cells were then washed with PBS, trypsinized and exposed to sonodynamic and photodynamic treatment as previously described. ROS production was assessed at 1, 5, 15, 30 and 60 min after treatments, and 10,000 events were considered for analysis by the FL1 channel (λ_ex_ 532 nm). ROS production was expressed as the integrated mean fluorescence intensity (iMFI), which was calculated as the product of the mean fluorescence intensity of the cells and the frequency of ROS-producing cells. The iMFI ratio was then calculated in order to yield the ratiometric increase in fluorescence per time point related to the iMFI of untreated cells, i.e., control cells [20].

#### 2.10.2. Mitochondrial Membrane Potential

The effects that sonodynamic treatment had on HT-29 or HDF 106-05 cell mitochondrial membrane potential was investigated using the Membrane Potential Detection Kit (BD Bioscience, San Jose, CA, USA) by flow cytometry. Mitochondrial functionality is related to optimal vital cellular status. Changes in mitochondrial membrane potential, particularly depolarization, were reported to occur during many cellular processes, such as production of ROS, activation of apoptosis and necrosis. 5,5′,6,6′-tetrachloro-1,1′,3,3′-tetraethylbenzimidazolcarbocyanine iodide (JC-1) is a membrane-permeable lipophilic cationic fluorochrome that penetrates cells, and its fluorescence is a reflection of mitochondrial membrane potential (Δψ). The fluorescence emission spectrum of JC-1 is dependent on the status of Δψ. JC-1 excites at λ_ex_ 488–490 nm and can exist in two different states, aggregates (λ_em_ 590 nm) or monomers (λ_em_ 527 nm), each with a different emission spectrum; both states exhibit fluorescence by FL-1 channel (λ_em_ 532 nm, green) but aggregates are also detectable by the FL-2 channel (λ_em_ 585 nm, red). When live cells are incubated with JC-1, the JC-1 penetrates the plasma membrane and mitochondria membrane. In polarized membranes of functional mitochondria conditions JC-1, accumulates in aggregate form, conversely, in depolarized membranes of less functional mitochondria JC-1 leaks out of the mitochondria and accumulates into the cytoplasm in monomeric form.

Briefly, HT-29 or HDF 106-05 cells were incubated for 24 h with P-Pd 500 μM at 37 °C and then exposed to US, as previously described. Moreover, a positive control was performed by exposing cells to 500 μM H_2_O_2_ for 3 h. JC-1 aggregates and monomers were investigated for each condition, and 10,000 events were recorded by the FL1 channel (λ_em_ 532 nm) and the FL2 channel (λ_em_ 585 nm); compensation was required due to fluorophore monomers and aggregates with partially overlapping emission spectra. Regions were placed around HT-29 or HDF 106-05 cell populations with high JC-1 aggregates and high monomer concentration (functional mitochondria); other regions were placed around HT-29 or HDF 106-05 cell populations with low JC-1 aggregates and high JC-1 monomer concentration (less functional mitochondria). For quantitative analysis, Δψ was expressed as the ratio between JC-1 aggregate and monomer mean fluorescence emission.

### 2.11. Membrane Fluidity/Phospholipid Polarization

The influence that US exposure had on HT-29 or HDF 106-05 cell membrane fluidity/phospholipid polarization was investigated by 1,6-diphenyl-1,3,5-hexatriene (DPH) fluorescence anisotropy using a polarized fluorescence spectrometer (LS 55 PerkinElmer, USA). DPH powder (MW = 232.32 g/mol) was dissolved to a final concentration of 2 mM in tetrahydrofuran (Sigma-Aldrich). Then, the pH of DPH final working solution (final concentration of 2 μM DPH in PBS) used for cells was measured and a value of pH 7.00 was obtained. HT-29 or HDF 106-05 cells were unexposed or exposed to US, as previously described. Positive controls for the reduction in anisotropy (increase in membrane fluidity and polarization) were obtained by exposing cells at 45 °C for 30 min [21,22]. Immediately after treatments, cells were incubated with working solution (2 μM DPH) at room temperature and protected by light. DPH anisotropy was evaluated at 30 and 90 min after being treated five times. Data are expressed as mean values ± standard deviation. The excitation wavelength and the detection wavelength were set at λ_ex_ 365 nm and λ_em_ 420 nm, respectively, and the excitation and emission split widths were set at 5 nm and 16 nm, respectively. Anisotropy (r) was calculated using the following equation:

(1) $$r=\frac{I_{VV\;}-GI_{VH}}{I_{VV}+2GI_{VH}}$$

where *I* is the fluorescence emission intensity and subscript *V* and *H* represent the vertical and horizontal excitation and emission polarized light orientations. *G* is a grating factor that accounts for differences in sensitivity to horizontal and vertical polarized light and is calculated as *G* = *I_HV_*/*I_HH_* [23]. In particular, membrane fluidity/phospholipid polarization was expressed as DPH fluorescence anisotropy.

### 2.12. Statistical Analysis

Data are shown as mean values ± standard deviation of three independent experiments. Statistical analyses were performed using Prism 7.0 software (GraphPad, La Jolla, CA, USA). According to the design of the experiment under analysis, multiple *t*-tests, two-way ANOVA, one-way ANOVA and Bonferroni’s test were used to calculate the threshold of significance. The statistical significance threshold was set at *p* < 0.05.

## 3. Results

### 3.1. Pd-P Uptake in HT-29 and HDF 106-05 Cells

**Figure 1:**
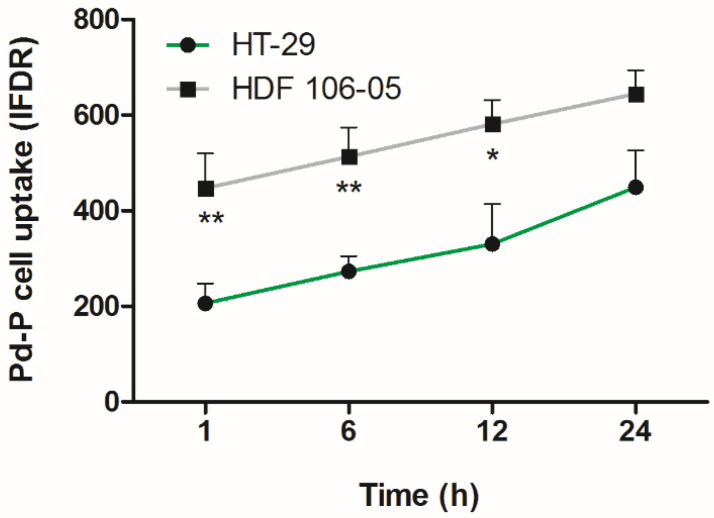
Pd-P cellular uptake in HT-29 and HDF 105-06 cells over time. HT-29 and HDF 105-06 cells were incubated for 1, 6, 12 and 24 h with 500 μM Pd-P, and stained with DAPI. Sample fluorescence intensity was determined using a fluorescent plate reader and expressed as intensity of fluorescence DAPI related (IFDR). Statistically significant difference versus HT-29 cells: * *p* < 0.05, ** *p* < 0.01.

In order to evaluate the best suitable time to perform sonodynamic and photodynamic treatment, HT-29 and HDF 105-06 cells were incubated with 500 µM Pd-P complex, and the relative intracellular fluorescence intensity was evaluated as IFDR. As shown in Figure 1, in HT-29 and HDF 106-05 cells, the porphyrin uptake increased over time, up to a maximum level at 24 h and, in the HDF 106-05 cells, porphyrin uptake was statistically significantly higher compared to HT-29 over time, up to 12 h. Moreover, at 24 h, the porphyrin uptake was not significantly different between the two cell lines. Therefore, for subsequent sonodynamic and photodynamic experiments, 24 h was selected as the suitable time for the Pd-P incubation, representing the best level of sensitizer uptake for both HT-29 and HDF 105-06 cells.

**Figure 2:**
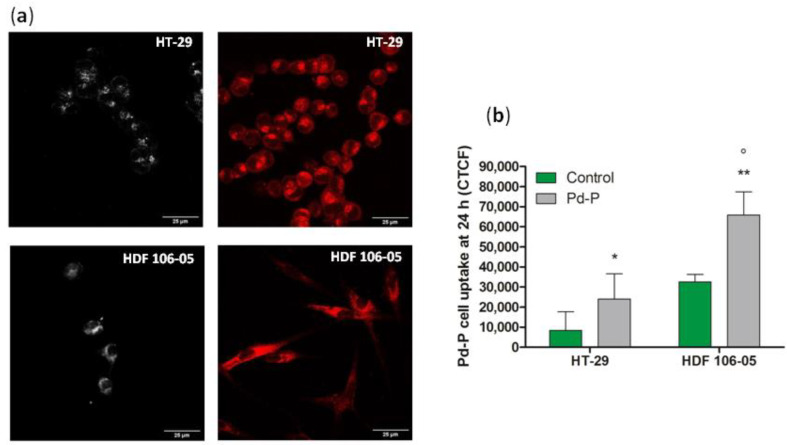
Pd-P cellular localization and quantification in HT-29 and HDF 105-06 cells after 24 h incubation. (**a**) Representative confocal fluorescence images of HT-29 and HDF 106-05 cells at 40× magnification (scale bar 25 μm): untreated HT-29 cells (bright fluorescence), HT-29 cells incubated for 24 h with 500 μM of Pd-P (red fluorescence), untreated HFD 106-05 cells (bright fluorescence) and HFD 106-05 cells incubated for 24 h with 500 μM of Pd-P (red fluorescence). (**b**) The confocal fluorescence quantification at 24 h is expressed as corrected total cell fluorescence (CTCF). Statistically significant difference versus untreated cells: * *p* < 0.05, ** *p* < 0.01. Statistically significant difference of HDF 106-05 cells incubated with Pd-P versus HT-29 cells incubated with Pd-P: ° *p* < 0.05.

Furthermore, to establish the cellular localization of the metal-porphyrin complex, a confocal fluorescence analysis of 500 µM P-Pd in HT-29 and HDF 106-05 cells was performed after 24 h of incubation. As shown in Figure 2a, Pd-P principally showed a cytoplasmic distribution. In particular, in HDF 106-05 cells, more fluorescent aggregates were visible at the cytoplasmic level and throughout the cellular body, whereas in HT-29 cells, more fluorescent aggregates were visible in the perinuclear regions. Finally, from confocal fluorescence analysis, we were also able to confirm that the concentration of the Pd-P complex in both cell lines, after 24 h incubation of the Pd-P complex, was statistically significantly different compared to the basal autofluorescence of untreated cells by CTCF quantification (Figure 2b).

### 3.2. Effect of Sonodynamic and Photodynamic Treatment on Co-Cultured HT-29 and HDF Cells

**Figure 3:**
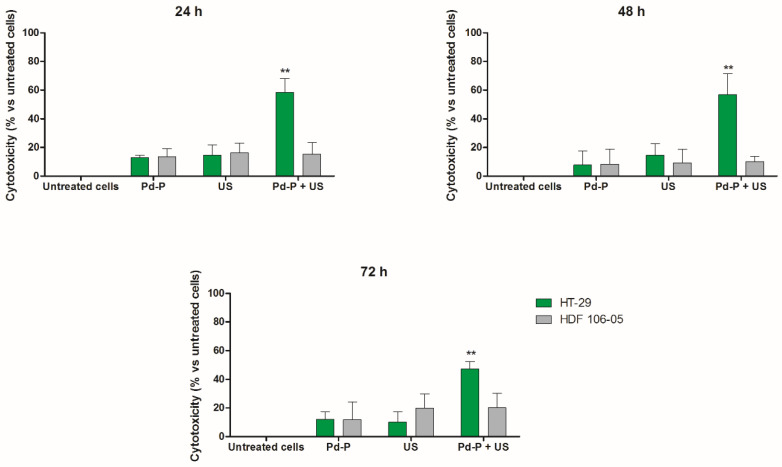
Effect of sonodynamic treatment on HT-29/HDF 106-05 co-culture. HT-29 and HDF 106-05 cells, in a 1:1 co-culture, were incubated for 24 h with Pd-P (500 μM), and then exposed to US (1.5 W/cm^2^ at 1.866 MHz, for 5 min). Cytotoxicity was then evaluated after 24, 48 and 72 h by counting HT-29 and HDF 106-05 cells. Data are expressed as percentage of cytotoxic treated cells versus untreated condition. Statistically significant difference versus untreated cells: ** *p* < 0.01.

Considering our previous results on the different responsiveness of cancer HT-29 and normal HDF 106-05 cells when separately subjected to sonodynamic treatment [12], we decided to investigate the Pd-P activation by US on a co-culture model between these two cell lines. Observing Figure 3, a significant increase in cytotoxicity was observed only on HT-29 cells when the cells in co-culture were incubated with 500 µM Pd-P and exposed to US (*p* < 0.01), compared to untreated co-culture, co-culture treated with the Pd-P alone or exposed only to US. Indeed, no statistically significant cytotoxicity was observed on HDF 106-05 cells in the co-culture model. These data demonstrated that even if cancer cells and normal cells were treated simultaneously in a co-culture, they preserved a different responsiveness over time to the sonodynamic treatment, similar to that observed when they were treated as separate cell lines [12].

Since oxidative stress is the main mechanism responsible for SDT-induced cytotoxicity, to gain further insight on the reason why HT-29 and HDF 106-05 cell lines showed a different response to SDT (Figure 3), the basal level of reduced glutathione—the major intracellular antioxidant system—was investigated in each cell line.

**Figure 4:**
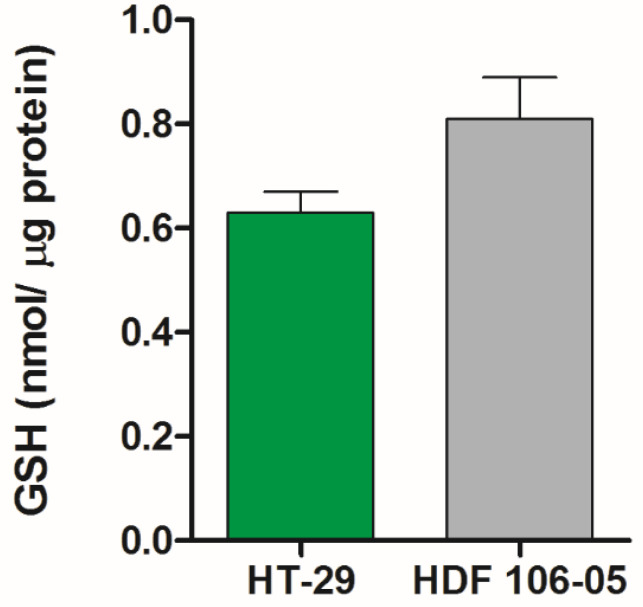
Intracellular GSH levels according to cell type. The total glutathione level of HT-29 and HDF 106-05 cells at a basal level, i.e., untreated cells, was analyzed using the Glutathione Assay Kit (Sigma-Aldrich) according to the manufacturer’s instructions and the GSH content (nmol) was normalized to the protein content (μg) of the sample.

As shown in Figure 4, no statistically significant difference in intracellular GSH levels was observed between HT-29 (0.63 ± 0.04) and HDF 106-05 (0.81 ± 0.08) cells, suggesting a similar role of the glutathione system in defense against oxidative stress in both cell lines.

**Figure 5:**
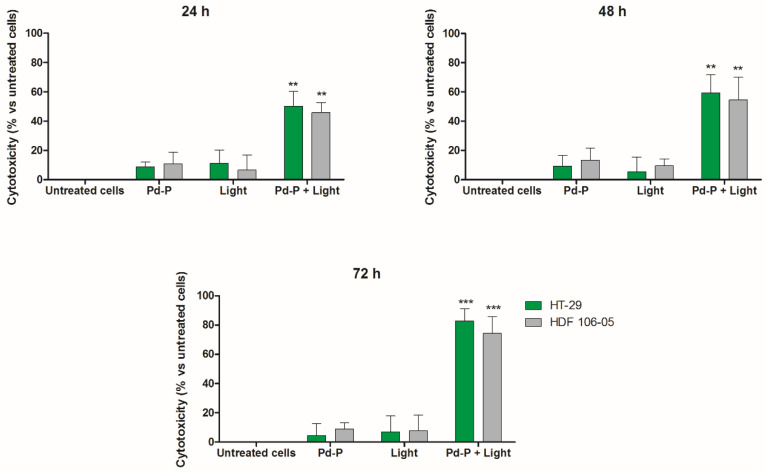
Effect of photodynamic treatment on HT-29/HDF 106-05 co-culture. HT-29 and HDF 106-05 cells, in a 1:1 co-culture, were incubated for 24 h with Pd-P (500 μM), and then exposed to light (15 mW/cm^2^ at 405 nm, for 5 min) at the appropriate wavelength (405 nm). Cytotoxicity was then evaluated after 24, 48 and 72 h by counting HT-29 and HDF 106-05 cells. Data are expressed as percentage of cytotoxic treated cells versus untreated condition. Statistically significant difference *versus* untreated cells: ** *p* < 0.01, *** *p* < 0.001.

Of note, when the HT-29 and HDF 106-05 co-culture underwent photodynamic treatment at the appropriate light wavelength, we observed significant cytotoxicity in both cell lines in co-culture compared to untreated co-culture, co-culture treated with Pd-P alone and exposed only to the light (Figure 5).

### 3.3. Effect of Sonodynamic and Photodynamic Treatment on ROS Production

**Figure 6:**
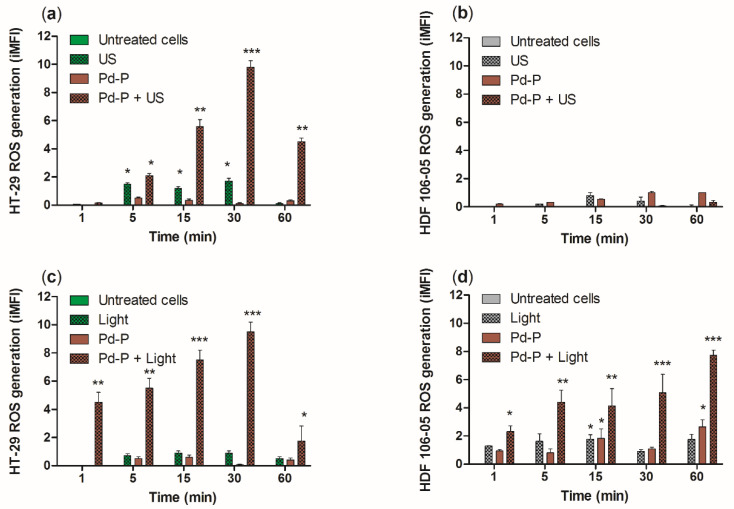
Reactive oxygen species production after sonodynamic or photodynamic treatment. HT-29 (**a**,**c**) and HDF 106-05 (**b**,**d**) cells were incubated for 24 h with 500 μM Pd-P and then exposed to US (1.5 W/cm^2^ at 1.866 MHz, for 5 min) or to light (15 mW/cm^2^ at 405 nm, for 5 min) at the appropriate wavelength (405 nm). ROS levels were determined at different time points after each treatment (1, 5, 15, 30 and 60 min) with 2′,7′-dichlorofluorescein diacetate (DCF-DA) assay using flow cytometry. Results are expressed as the integrated mean fluorescence (iMFI) ratio. Statistically significant difference versus untreated cells: * *p* < 0.05, ** *p* < 0.01, *** *p* < 0.001.

Since it has been shown that not only photodynamic but also sonodynamic-mediated porphyrin activation leads to significant intracellular ROS production [24,25,26,27], the ROS generation in HT-29 and HDF 106-05 was evaluated after sonodynamic or photodynamic treatment compared to untreated cells and cells exposed only to US or light by using the DCFH-DA cytofluorimetric assay. In HT-29 cells, a significant increase in ROS was observed after 5 (*p* < 0.05), 15 (*p* < 0.01) and 30 (*p* < 0.001) min from the SDT, with a slight decline at 60 min (*p* < 0.01) compared to untreated cells (Figure 6a). A small but statistically significant increase in ROS production was observed after 5 (*p* < 0.05), 15 (*p* < 0.05) and 30 (*p* < 0.05) min from the US exposure alone compared to untreated cells; this ROS generation was not able to induce cell damage as shown in Figure 3. In HDF 106-05 cells, no ROS production was observed over time after each single treatment, and even after SDT (Figure 6b), demonstrating an important difference in ROS production between cancer cells and normal cells, mirroring the cytotoxicity data (Figure 3).

Conversely, after PDT, the maximum in ROS production was observed at 30 min in HT-29 cells (Figure 6c, *p* < 0.001) and at 60 min in HDF-106 cells (Figure 6d, *p* < 0.001). The PDT-mediated ROS production is therefore in line with the PDT cytotoxicity observed in both cell lines (Figure 5). These results lead to the suggestion that the main difference between SDT and PDT in HT-29 and HDF-106 cytotoxicity and ROS generation could be ascribed to specific cell structures, such as plasma membrane and mitochondria, due to the different physical nature of the waves employed, electromagnetic and mechanical, respectively.

### 3.4. Effects of US Exposure on Cytoplasmic Cell Membrane Fluidity

Considering the different behavior of cancer HT-29 and normal HDF 106-05 cells exposed to SDT in co-culture, and the possible role played by differences in their cytoplasmic cell membrane in eliciting, under US exposure, the intramembrane cavitation according to the BLS theory [16], the plasma membrane fluidity of each cell line before and after US exposure was investigated. This study was carried out to support our hypothesis that, in the presence of differences in mechanical properties of the plasma membrane, a different development of intramembrane cavitation can take place. Accordingly, the resulting modification in the US-induced energy transfer across the plasma cell membrane can lead to differences in the sonosensitizer activation.

**Figure 7:**
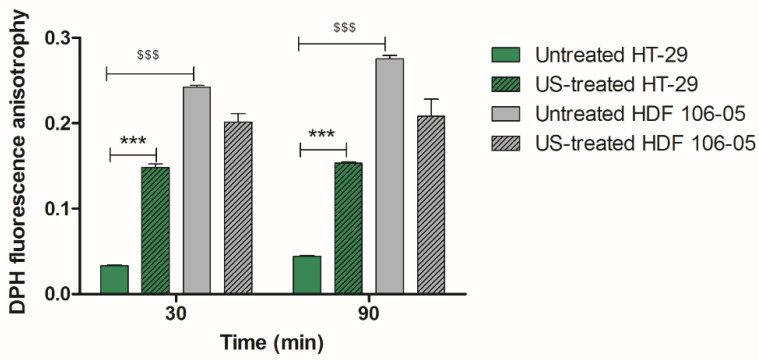
Effects of US exposure on cell membrane fluidity. HT-29 and HDF 106-05 cells were unexposed or exposed to US (1.5 W/cm^2^ at 1.866 MHz, for 5 min). Membrane fluidity/polarization was evaluated at different time points after the treatment (30 and 90 min) by the DPH (1,6-diphenyl-1,3,5-hexatriene) fluorescence anisotropy assay using a polarized spectrophotometer. Membrane fluidity/polarization was expressed as DPH fluorescence anisotropy. Statistically significant difference of untreated HDF 106-05 versus untreated HT-29 cells: ^$$$^ *p* < 0.001. Statistically significant difference *versus* untreated cells: *** *p* < 0.001.

Cell membrane fluidity was investigated by evaluating the fluorescence anisotropy of 1,6-diphenyl-1,3,5 hexatriene (DPH), a lipid probe which stains the hydrophobic core of the cell membrane. Briefly, when a significant decrease in DPH fluorescence anisotropy is measured, a substantial increase in membrane fluidity occurs, indicating that cell membrane lipid chains are more lax. Figure 7 shows a statistical difference in fluorescence anisotropy between the two cell lines, with a higher DPH fluorescence anisotropy in HDF 106-05 compared to HT-29 cells at basal/control conditions, i.e., when cells are untreated (*p* < 0.001). This means that the two cell lines have different starting cell membrane conditions, with the cell membrane of cancer cells being more fluid compared to the one of normal cells. The observed strong modification (*p* < 0.001) in the membrane fluidity of HT-29 under US exposure, compared to that observed in HDF-106 cells, suggests a significant change in the HT-29 cytoplasmic cell membrane, which could support a different intramembrane cavitation development under US exposure of the two cell lines.

### 3.5. Effects of Sonodynamic Treatment on Mitochondrial Function

**Figure 8:**
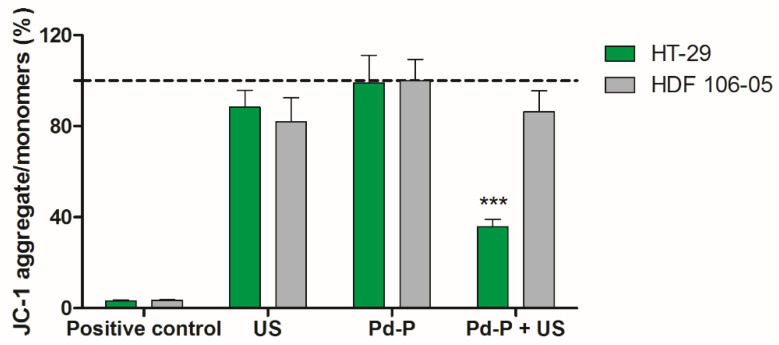
Cell mitochondrial membrane potential after sonodynamic treatment. HT-29 and HDF 106-05 cells were incubated for 24 h with 500 μM Pd-P and then exposed to US (1.5 W/cm^2^ at 1.866 MHz, for 5 min). Mitochondrial membrane potential was evaluated by JC-1 assay immediately after treatment by flow cytometry and expressed as percentage of JC-1 aggregates to monomers fluorescence ratio in each sample. A positive control was obtained by exposing cells to H_2_O_2_ (500 μM) for 3 h. The mitochondrial membrane potential of untreated cells is represented by the dashed line. Statistically significant difference versus untreated cells: *** *p* < 0.001.

The different behavior between HT-29 and HDF 106-06 cells in co-culture under SDT may be caused by dissimilarity in the US-induced energy transfer across their plasma membrane resulting from the observed different plasma membrane fluidity. This phenomenon could also influence the sonosensitizer activation and, accordingly, mitochondria, which play a central role for a variety of cellular processes such as intracellular ROS generation and Ca^2+^ signaling [28] and are a key element for the US-mediated sonosensitizer activation and the SDT efficacy [29]. To this end, the mitochondrial functionality as mitochondrial membrane potential was investigated in both cell lines immediately after the sonodynamic treatment. The cytofluorimetric JC-1 assay on HT-29 cells showed an increase in fluorescent monomeric forms and a subsequent significant reduction in the aggregates-to-monomers ratio, due to a mitochondrial membrane potential reduction when cells underwent sonodynamic treatment (Figure 8, 35.8 ± 3.2% *p* < 0.001). However, when HDF 106-05 cells were exposed to the same sonodynamic treatment, no difference in the JC-1 aggregates/monomers was observed (Figure 8, 86.3 ± 9.2%), highlighting a mitochondrial function similar to that of untreated cells. Therefore, this result supports our hypothesis that a connection is present between plasma membrane fluidity and US-induced energy transfer across the plasma membrane. In other words, differences between cell lines in plasma membrane fluidity could be responsible for differences in cellular responses to SDT, such as intracellular sonosensitizer activation, ROS production, mitochondrial membrane potential reduction and cell death.

## 4. Discussion

One of the main goals of anticancer drug therapy is to determine the selective destruction of cancer cells while leaving normal cells unaffected. Indeed, the poor drug selectivity towards cancer cells is one of the main causes of severe side effects associated with cytotoxic chemotherapeutic drugs [30]. Developing targeted therapies such as small molecule inhibitors and antibody targeted therapies has significantly improved the specificity of cancer treatment [31], but improvement can also be achieved by studying US-based treatment that takes advantages of structural differences between cancer cells and normal cells [14]. To this end, the use of US to activate intracellular sonosensitizer, in the so-called sonodynamic treatment, could be very promising. Sonodynamic treatment works with low intensity US, which avoids temperature increases in the target site, to trigger the cytotoxic nature of sensitizers such as porphyrins [32]. Porphyrins are well-known photosensitizers as their tetrapyrrole ring structure can be activated by light to generate ROS, causing damage to cell structures and cell death [33]. These features also make porphyrins also suitable sonosensitizers, i.e., compounds responsive to US, due to the hypothesis of the sonoluminescence occurrence under appropriate US exposure [34].

In order to investigate if the sonodynamic treatment could be a selective cancer treatment, a co-culture of human colon cancer HT-29 and fibroblast HDF 106-05 cells was developed. The palladium (II) porphyrin complex (Pd-P) was chosen as the sonosensitizer as it is well-accepted that the anticancer efficacy of sonodynamic treatment relies on the induction of a strong ROS generation due to the US-mediated activation of the sonosensitizer. Indeed, we previously showed that the insertion of Pd (II) in the porphyrin rings results in highly efficient ROS production under US exposure [12].

Before investigating the possible differences in response to the SDT by the two cell lines considered in co-culture, i.e., HT-29 and HDF 106-05, Pd-P uptake by the two cell lines was investigated in order to establish the optimal incubation time to perform SDT. Therefore, HT-29 and HDF 106-05 cells were incubated with the same noncytotoxic Pd-P concentration at different time points, and intracellular fluorescence quantification was performed, showing similar Pd-P uptake after 24 h of incubation in both cell lines (Figure 1). Interestingly, focusing on confocal images (Figure 2), it was possible to note a preferential Pd-P localization in the perinuclear regions of HT-29 cell cytoplasm, while Pd-P was localized all over the cellular body in the HDF 106-05 cell cytoplasm. Since it has been reported that in cancer cells, mitochondria are mostly localized at a perinuclear level, whereas in normal cells, they are spread all over the cellular body [35,36], and it has been considered that porphyrins accumulate preferentially in mitochondria [37], we suggested that the Pd-P complex preferentially accumulated in the mitochondria in both cell lines.

Following the results of the Pd-P uptake experiments in our cell lines, SDT was carried out in a co-culture of HT-29 and HDF 106-05 cells (1:1), showing a very significant cytotoxicity over time on HT-29 cells, but not on HDF 106-05 cells (Figure 3). To avoid any misinterpretations in our cytotoxic results between HT-29 and HDF 106-05 cells in co-culture, due to a possible difference in responses to the oxidative stress trigger by SDT, the amount of intracellular GSH was investigated. Indeed, cells can have a common antioxidant defensive strategy against ROS action, and one of the most important is the GSH redox cycle, able to metabolize hydrogen peroxide and to minimize its participation in reactions leading to hydroxyl radical formation [38,39]. In HT-29 and HDF 106-05 cell lines, no statistically significant difference in the intracellular amount of GSH was observed, suggesting that the response of the GSH system to oxidative stress could be similar in both the considered cell lines (Figure 4) and, most importantly, that our different cytotoxic results between HT-29 and HDF 106-05 cells in our co-culture model did not appear to be affected by the amount of intracellular GSH.

To understand the different cytotoxicity observed between HT-29 and HDF 106-05 cells in co-culture after the sonodynamic treatment, we investigated the cytotoxic effect of a photodynamic treatment with the same sensitizer, Pd-P, on the same HT-29 and HDF 106-05 co-culture. Indeed, SDT was developed as a novel, promising non-invasive anticancer approach derived from PDT and, furthermore, it is well known that the low selectivity of PDT towards malignant cells is mainly due to the direct transmission of light energy to the sensitizer without involving structures or intracellular organelles that could be different among cells, such as the outer cell membrane [40]. Therefore, after 24 h of incubation of a noncytotoxic concentration of Pd-P in both cell lines, our HT-29 and HDF 106-05 co-culture was subjected to PDT, and a significant cytotoxicity over time in both cancer HT-29 and normal HDF 106-05 cell lines was observed (Figure 5). To confirm these findings, the ROS production in our co-culture was investigated both after STD and PDT, since oxidative stress is the main mechanism underlying the cytotoxic effect of both treatments. ROS were strongly generated in HT-29 cells, but not in HDF 106-05 cells when our co-culture was subjected to sonodynamic treatment, whereas a strong ROS generation in both cell lines was observed when our co-culture was subjected to PDT (Figure 6), mirroring the cytotoxicity data. These data are in line with other reports confirming the strong ROS production as the pivotal step in the sonodynamic and photodynamic cytotoxic effect [8,41].

Since the major difference between SDT and PDT is the energy source used to activate the sensitizer (US versus light), this discrepancy between the SDT and PDT results suggested that US and light could activate the intracellular sensitizer differently in HT-29 and HDF 106-05 cells, probably due to the diverse nature of the wave, mechanical and electromagnetic, and therefore a different mechanism and amount of energy deposited to the sensitizer. Specifically, our findings suggested that, in SDT, the US-mediated intracellular sensitizer activation could be more influenced by differences in the cell structure of HT-29 and HDF 106-05 cells compared to PDT because, due to the type of energy released from the light, the selectivity of photodynamic action can be better achieved investigating cell organelles-targeted photosensitizers instead of cell structure differences between normal and cancer cells [42].

In order to understand whether the cell structure plays a role in the selective cytotoxicity towards cancer cells during SDT, plasma membrane fluidity and mitochondrial membrane potential in both cell lines were investigated. Our choice to initially focus our attention on the plasma cell membrane was based on strong evidence that malignant and normal cells differ not only in their metabolism and morphology, but also in the plasma cell membrane architecture. This latter aspect can lead to differences in terms of mechanical properties between cancer cells and normal cells, being crucial for the success of the sonodynamic approach, as the sonosensitizer intracellular activation is mediated by an US-induced energy transfer across the plasma cell membrane [43,44,45]. Indeed, one of the main interesting hypotheses explaining the US-induced energy transfer across the cell membrane to activate the intracellular sonosensitizer is represented by the bilayer sonophore (BLS) theory. According to this theory, Krasovitski et al. suggested that the cytoplasmic membrane, under appropriate conditions, is capable of transforming the US oscillating acoustic pressure wave into an intramembrane cavitation, which could explain all US-induced bioeffects [16]. In other words, this cyclic expansion and contraction of the BLS could stimulate cycles of stretch and release in the plasma membrane and in the cytoskeleton, becoming a source of intramembrane cavitation. Therefore, we suggest that this kind of cavitation could generate intracellular submicron-sized gas bubbles that, when collapsed, release very high energy and perhaps also sonoluminescence [34].

Therefore, our hypothesis was that the SDT selective cytotoxicity between HT-29 and HDF 106-05 cells could be based on BLS occurrence, thanks to our US set-up and according to the different mechanical properties of the plasma membrane of the two cell lines considered. Therefore, to investigate our hypothesis, we studied an important plasma membrane feature that could affect, according to BLS hypothesis, the US-induced energy transfer across the plasma membrane namely, the cell membrane fluidity. Cell membrane fluidity was analyzed by using DPH, a lipid probe that is able to link phospholipid chains contained in the cytoplasmic membrane bilayer, where an increase in DPH fluorescence anisotropy occurs in the presence of destabilized phospholipid polarization, being related to increased cell membrane fluidity [22,46]. Specifically, HDF 106-05 normal cells at basal conditions, i.e., without US exposure, showed a significantly lower membrane fluidity compared to HT-29 cancer cells, with normal cells being stiffer than cancer cells (Figure 7).

These data are in agreement with other studies that reported cancer cells as being more compliant to physical stimuli such as acoustic waves than normal cells, and this evidence could support the preferential response of cancer cells to sonodynamic treatment [14,47]. Indeed, some researchers demonstrated that normal cells and cancer cells display differences in cell–cell interactions, cytoskeleton organization and subcellular structure, determining a specific elasticity and, therefore, distinct mechanical phenotypes [44,45,47]. Indeed, cancer cells, in general, appear softer than their normal counterparts, while conversely, normal cells show greater stiffness and higher resistance to mechanical stress compared to cancer cells [13,14,43]. Therefore, the observed difference in cell membrane fluidity between the two cell lines under investigation could be related to the selective response of cancer cells to the sonodynamic treatment. The difference highlighted between cancer cells and normal cells in their responsiveness to sonodynamic treatment is also supported by Kosheleva’s work, where it was noticed that the synergistic cytotoxic effects of US and nanoparticles were more pronounced in A549 lung cancer cells than in their normal counterparts, BEAS-2B cells [48]. Moreover, after US exposure, we observed a decrease in membrane fluidity for HT-29 (Figure 7), suggesting a bilayer cell membrane separation, as described in Di Giacinto’s work [49]. Therefore, our results could support the hypothesis that SDT cytotoxic selectivity between HT-29 and HDF 106-05 cells may be based on the BLS theory thanks to our US set-up, and according to a different mechanical property of the plasma membrane between the two cell lines.

Another important aspect that can elucidate and highlight the differences between cancer cells and normal cells under SDT is related to the effect of SDT on HT-29 and HDF 106-05 mitochondria, since mitochondria are one of the main ROS mediators, playing a pivotal role in controlling process that can lead to cell death [50]. Furthermore, mitochondria are an important subcellular target for sensitizers, and are also sensitive to US exposure [27,51,52,53]. Therefore, mitochondrial membrane potential was investigated, and a significant mitochondrial membrane potential reduction, along with a subsequent mitochondria dysfunction, was only observed when cancer HT-29 cells underwent sonodynamic treatment (Figure 8). These data are in line with the sonodynamic-induced cytotoxicity in HT-29 cells and the inefficacy of this treatment in inducing cytotoxicity in HDF 106-05 cells. Therefore, the different SDT responses between the two cell lines in our co-culture model could be also ascribed to a different effect on mitochondria, in line with diverse sensitizer activation under US exposure, occurring in cancer cells and not in normal cells, in accord with the BLS theory.

## 5. Conclusions

Cancer cells and normal cells are considerably different in cell structure and mechanical properties. Our data highlight a marked difference between cancer cells and normal cells in term of responsiveness to the sonodynamic treatment, with US being able to selectively trigger the cytotoxicity of the intracellular sonosensitizer on cancer cells only. This different behavior between cancer cells and normal cells that is responsible for different US-induced bioeffects can be ascribed to diversity in plasma membrane properties such as membrane fluidity according to the BLS theory. The observed significant lower stiffness of cancer cells compared to normal cells could allow an efficient US energy transfer to the intracellular sonosensitizer in mitochondria in cancer cells only, overcoming one of the main drawbacks in cancer therapy, namely the selectivity. Therefore, plasma membrane properties could influence the sonodynamic treatment despite what has been observed in photodynamic treatment, increasing the interest in exploiting SDT as a significant advancement in the battle against cancer.

## Data Availability

The data presented in this study are available on request from the corresponding author.
